# A unique neuropsychophysiological approach to objectify emotion (dys)regulation in healthy older adults during the COVID-19 pandemic

**DOI:** 10.1038/s41598-023-50310-1

**Published:** 2023-12-28

**Authors:** Martina Amanzio, Giuseppina Elena Cipriani, Nicola Canessa, Francesca Borghesi, Alice Chirico, Pietro Cipresso

**Affiliations:** 1 Department of Psychology, University of Turin, 10024 Turin, Italy; 2 ICoN Center, Scuola Universitaria Superiore IUSS, 27100 Pavia, Italy; 3 Cognitive Neuroscience Laboratory of Pavia Institute, Istituti Clinici Scientifici Maugeri IRCCS, 27100 Pavia, Italy; 4 Department of Psychology, Research Center in Communication Psychology, Università Cattolica del Sacro Cuore, 20123 Milan, Italy; 5 Applied Technology for Neuro-Psychology Laboratory, Istituto Auxologico Italiano, Istituto di Ricovero e Cura a Carattere Scientifico, 20145 Milan, Italy

**Keywords:** Cognitive ageing, Emotion

## Abstract

The response of older people to the COVID-19 pandemic has attracted much attention as they are at increased risk of adverse outcomes. A longitudinal study has shown that improvement in global cognitive, executive and language functioning in healthy older adults enrolled at the University of the Third Age appears to play a protective role against emotional dysregulation and mood changes during the pandemic. To date, no study has examined emotional dysregulation through COVID-19-related images using facial electromyographic recordings in healthy older adults. Therefore, we aimed to analyze the relationships between zygomaticus and corrugator reactivity, neuropsychological measures, and the affective dimensions of arousal, dominance, and valence. The results showed an unexpected association between higher zygomaticus activity and higher levels of apathy, depression, and anxiety. In contrast, increased contracture of the corrugator was associated with poorer performance on cognitive tests (global cognition, memory, executive functions) and physical status, i.e., walking speed. These results are consistent with the reappraisal of emotional stimuli in response to the challenges of the pandemic. Interestingly, COVID-19-related stimuli triggered the activation of bottom-up affectivity strategies associated with higher mood levels and interacted with top-down factors that play an important role in the dysregulation of cognitive control.

## Introduction

The study of healthy older adults represents a valuable opportunity to objectify the determinants of early mild changes by examining their responses during the COVID-19 pandemic, which may be crucial for planning interventions in future health emergencies.

To date, few studies have used a longitudinal approach to examine healthy older people by comparing data before and during the pandemic^1–3^.

The first study showed how mood swings, executive attention and physical condition played an important role during the COVID-19 pandemic. In particular, lockdown fatigue triggered by the restrictive COVID-19 measures was associated with higher depression scores and reduced attentional processing and walking speed^1^.

Furthermore, fear of SARS-CoV-2 infection could be an additional source of concern for this population, increasing anxiety and thus affecting quality of life^4^. Notably, longitudinal data have indeed shown that the perceived threat of SARS-CoV-2 infection was predicted by physical, cognitive and mood-related baseline factors, specifically a combination of prefrailty status, language comprehension performance and slower information processing, and anxiety^2^.

Nevertheless, a recent study reported the positive responses of older adults to such a negative scenario, in terms of comparable cognitive performance (e.g., memory and attention) and physical condition before and after the pandemic^3^. Surprisingly, participants who attended courses at the University of the Third Age even showed an improvement in global cognitive, executive and language performance. However, a change in mood was observed after the lockdown, especially in terms of apathy and anxiety. To investigate emotional dysregulation, participants’ heart rate variability (HRV) was recorded while they were shown pandemic-related images. The results suggested that preserved global cognition played a protective role against the negative effects of the pandemic on mood and emotional (dys)regulation^3^.

To further uncover the reactions of older people during the pandemic, and in particular the relationship between cognitive function, mood, physical state and emotional (dys)regulation, we used a novel interdisciplinary approach. Namely, we combined neuropsychological data with facial muscle reactivity through electromyographic recordings (fEMG) during the presentation of images associated with the early—and most stressful—phase of the pandemic, which is strategic given the ongoing impact. fEMG represents an interesting way to better understand and objectify the emotional impact of the pandemic on healthy older adults who have experienced this stressful event. It is non-invasive, and yet sensitive to transient and subtle changes in facial muscle reactivity in an ongoing emotional process when visual observation is unavailable or unclear^5,6^. In other words, the fEMG is a useful tool to infer a person’s affective state and mood^7,8^. It has been widely used to study emotional responses elicited by affective visual stimuli (e.g.,^5,9–11^), especially based on the three-dimensional model of emotion (pleasure, arousal, dominance), which provides useful descriptions of emotional states^12^. In particular, the reactivity of the corrugator supercilii and zygomaticus major has previously been associated with the expression of negative and positive emotions, respectively^13^, and with consistent subjective emotional experience^14^. Corrugator and zygomaticus contractures are able to effectively discriminate between arousal (intensity) and valence (pleasure) of an emotional state^15^ and with the implicit processing of negative and positive emotions, i.e. the reappraisal associated with frowning and smiling, respectively^16^.

On this basis, 35 healthy and socially active older adults underwent a thorough neuropsychological assessment approximately 20 months after the outbreak of the pandemic to collect data on (1) global cognition, memory, language, executive and attentional functions, (2) mood, and (3) physical condition. During the same period, we assessed the degree of their emotional (dys)regulation during the presentation of COVID-19-related images using fEMG reactivity and the Self-Assessment Manikin (SAM) metric^17^.

We therefore examined the correlation between the neuropsychological measures, the fEMG data and the affective dimensions of arousal, dominance and valence using SAM.

Considering that emotional self-regulation is crucial for well-being during stressful events^18^ and that fEMG can be intentionally modulated to serve emotion regulation^19^, we hypothesized that a pattern of facial reactivity will be useful to objectify the dysregulation of cognitive control, namely reappraisal in the face of the pandemic. The expected results may reveal strategies for processing emotional stimuli with higher demands on cognitive emotion regulation, where neuropsychological factors such as executive functions, memory and mood changes should play an important role in the dysregulation of cognitive control.

## Results

### Multidimensional neuropsychological battery

Participants’ cognitive, affective, and physical characteristics are listed in Table 1. All neuropsychological variables follow a normal distribution and have no missing values. All participants were right-handed (without having changed their dominant hand during their lifetime).

**Table 1:** Multidimensional neuropsychological battery.

	N	M	SD	Cut-off
Sociodemographic characteristics				
Subjects	35			
Gender				
Women	27			
Men	8			
Age [years]		70.40	5.58	
Education [years]		13.29	1.90	
Cognitive domain				
MMSE		29.34	0.80	≥ 23.8
ACE-R		94.26	2.89	≥ 79 (< 75 years old);
				≥ 60 (> 75 years old)
MoCA		26.20	2.64	≥ 17.363
CFI self-report		2.76	1.66	
CFI partner-report		1.54	1.39	
RMT—15 instant words		43.86	7.64	≥ 28.53
RMT—15 delayed words		9.63	3.11	≥ 4.69
TMT—part A		38.63	11.61	≤ 94
TMT—part B		95.89	38.84	≤ 283
TMT B-A		57.29	32.30	≤ 187
TT		34.77	1.03	≤ 29
Mood domain				
AES		10.46	6.70	≤ 14
BDI		6.60	5.89	≤ 9
HARS		7.83	5.15	≤ 14
Physical and health status domain				
Frailty phenotype status		0.40	0.60	0
Robust	23			
Pre-frail	12			
Frail	0			
CIRS				
Severity index		1.37	0.18	
Comorbidity index		1.46	0.98	

*N* number, *M* mean, *SD* standard deviation, *MMSE* Mini Mental State Examination, *ACE-R* Addenbrooke’s Cognitive Examination—Revised version, *MoCA* Montreal Cognitive Assessment, *CFI* Cognitive Functional Instrument. *RMT* Rey Memory Test, *TMT* Trail Making Test, *TT* Token Test, *AES* Apathy Evaluation Scale, *BDI* Beck Depression Inventory, *HARS* Hamilton Rating Scale for Anxiety, *CIRS* Cumulative Illness Rating Scale.

#### Sociodemographic characteristics and cognitive domain

In terms of socio-demographic characteristics, 77.14% of the participants were women and 22.86% were men, their mean age was 70.4 years (age range: 62–82), and their mean level of education was 13.29 years (range: 9–17). They attended middle school (N = 3), high school (N = 25), and college (N = 7).

40% of the sample lived alone and the remaining 60% lived with at least one person (in most cases their partner). According to the Hollingshead index (HI^20,21^), most of the subjects had a medium to high socioeconomic status (SES). More specifically, 8.57% of them fell in the highest status (HI range: 66–55), 51.43% in the second (HI range: 54–40), 37.14% in the third (HI range: 39–30), 2.86% in the fourth (HI range: 29–20), and none in the lowest (HI range: 19–8).

The subjects’ performance on both the Addenbrooke’s Cognitive Examination-Revised Version (ACE-R^22^) and the Mini Mental State Examination (MMSE^23^) did not indicate the presence of mild cognitive impairment (MCI, i.e.,^24^), as all subjects scored above the cut-off and did not report subjective cognitive decline (SCD^25^), as assessed by the Cognitive Function Instrument (CFI^26^). Their performance was above cut-off on the following tests: the Montreal Cognitive Assessment (MoCA^27^) (100%), Rey Memory Test (RMT^28^)—15 immediate words (97.14%), RMT—15 delayed words (94.29%), Trail-Making Test (TMT^29^)—part A (100%), TMT—part B (100%), TMT B-A (97.14%), and Token Test (TT^30^) (100%). Although some performances in the neuropsychological tests were below the cut-off value, the percentages corresponded to the error rate in the normative older population.

#### Mood domain

Some participants showed mood swings. In particular, higher scores for apathy (Apathy Rating Scale, AES^31^), depression (Beck Depression Inventory, BDI^32^), and anxiety (Hamilton Rating Scale for Anxiety, HARS^33^) were found in 37.14%, 28.57%, and 14.29% of the sample, respectively.

#### Physical and health status domain

Most of the participants were classified as “robust” (65.71%); the rest of the sample was prefrail (34.29%). On the other hand, none of the participants met Fried et al. inclusion criteria for frailty status^34^.

The subjects scored 1.37 ± 0.18 (mean ± standard deviation) on the Cumulative Illness Rating Scale (CIRS^35^)—severity index and 1.46 ± 0.98 (mean ± standard deviation) on the CIRS—comorbidity index.

Regarding the data on immunization against SARS-CoV-2, 20% of the participants reported having been vaccinated with the first dose, 74.29% with the second dose, and only two people (5.71%) had not been vaccinated.

Only one of the participants reported being infected with SARS-CoV-2, while the others stated that they did not have COVID-19.

### Correlation matrix between the multidimensional neuropsychological battery and the psychophysiological assessment

We used a correlation matrix to investigate the relationship between neuropsychological performance and emotional dysregulation as measured by the mean amplitude of the fEMG. Only significant correlations between fEMG-1 (corrugator activity) or fEMG-2 (zygomaticus activity) and the three domains of neuropsychological assessment were listed in Table 2. A negative correlation was found between increased corrugator activity and decreased performance in global cognition (ACE-R: r = − 0.429, *p* < 0.05) and long-term memory (RMT-15 delayed words: r = − 0.370, *p* < 0.05). A positive correlation was found between increased corrugator activity and higher time performance on tasks assessing (a) visual processing and attentional-executive (TMT—part A: r = 0.512, *p* < 0.01; TMT—part B: r = 0.521, *p* < 0.01; TMT B-A: r = 0.439, *p* < 0.05) and (b) walking speed (r = 0.482, *p* < 0.01).

**Table 2:** Correlation matrix.

	EMG-1	95% CI	EMG-2	95% CI
Addenbrooke’s Cognitive Examination—Revised version	r = − 0.429 *P* < 0.05	[− 0.673, − 0.100]		
Rey memory test—15 delayed words	r = − 0.37 *P* < 0.05	[− 0.633, − 0.031]		
Trail making test – part A	r = 0.512 *P* < 0.01	[0.204, 0.727]		
Trail making test – part B	r = 0.521 *P* < 0.01	[0.217, 0.733]		
Trail making test B-A	r = 0.439 *P* < 0.05	[0.113, 0.680]		
Frailty criterion -walking speed	r = 0.482 *P* < 0.01	[0.166, 0.708]		
Apathy evaluation scale			r = 0.444 *P* < 0.01	[0.118, 0.683]
Beck Depression Inventory			r = 0.689 *P* < 0.001	[0.453, 0.835]
Hamilton Rating Scale for Anxiety			r = 0.507 *P* < 0.01	[0.198, 0.724]

*EMG-1* electromyography – corrugator activity, *EMG-2* electromyography – zygomaticus activity, *CI* confidence interval (95% CI are indicated as the inferior and superior values of the interval), *r* Pearson’s correlation coefficient, *P* *p* value.

The results related to fEMG-2 showed significant correlations only with mood scales, i.e., AES (r = 0.444, *p* < 0.01), BDI (r = 0.689, *p* < 0.001), and HARS (r = 0.507, *p* < 0.01), suggesting that increased zygomaticus activity is associated with higher scores for apathy, depression, and anxiety, respectively.

Given the significant associations between the neuropsychological data and the fEMG, we created a new correlation matrix to further investigate the relationship between the affective dimensions assessed using SAM.

In Table 3, we first presented (a) the negative relationship between valence and arousal (r = − 0.603, *p* < 0.001), (b) the negative relationship between dominance and arousal (r = -0.414, *p* < 0.05), and (c) the positive relationship between valence and dominance (r = 0.497, *p* < 0.01).

**Table 3:** Correlation matrix between the mean values of Arousal, Dominance and Valence and neuropsychological data.

	Arousal	95% CI	Domi-nance	95% CI	Valence	95% CI
Arousal						
Dominance	r = − 0.414 *P* < 0.05	[− 0.657, − 0.094]				
Valence	r = − 0.603 *P* < 0.001	[− 0.779, − 0.337]	r = 0.497 *P* < 0.01	[0.196, 0.712]		
Trail Making Test – part B					r = 0.350 *P* < 0.05	[0.019, 0.612]
Trail Making Test B-A					r = 0.364 *P* < 0.05	[0.035, 0.622]
Apathy Evaluation Scale	r = 0.350 *P* < 0.05	[0.019, 0.612]	r = − 0.411 *P* < 0.05	[− 0.654, − 0.090]		
Beck Depression Inventory			*r* = − 0.42 P < 0.05	[− 0.661, − 0.101]		
Hamilton Rating Scale for Anxiety	r = 0.401 *P* < 0.05	[0.078, 0.647]	*r* = − 0.523 *P* < 0.01	[− 0.729, − 0.230]		

*CI* confidence interval (95% CI are indicated as the inferior and superior values of the interval), *r* Pearson’s correlation coefficient. *P* *p* value.

**Figure 1:**
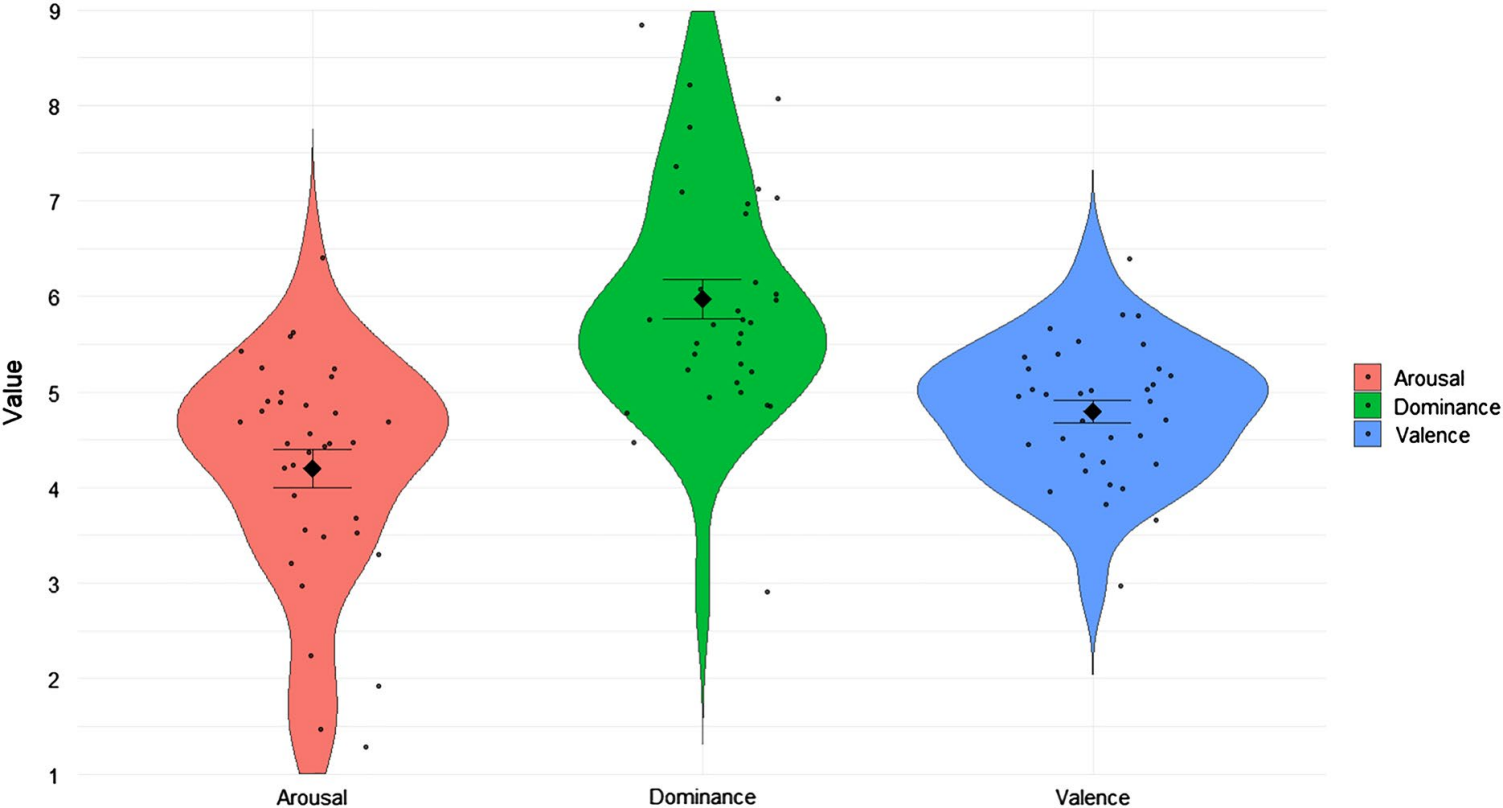
Mean (rhombus) ± standard deviation (lines) of mean ratings of Arousal (red), Dominance (green), and Valence (blue) of the Self-Assessment Manikin (SAM) on the 9-point Likert scale of all participants. Individual subjects (black circles) are also shown considering their mean SAM scores.

Figure 1 shows the means ± standard deviations of the presented images in terms of arousal, dominance, and valence for all participants together with the individual mean values of SAM. On the 9-point Likert scale of SAM, we observed a higher mean for dominance (M = 5.97 ± SD = 1.21), followed by valence (M = 4.8 ± SD = 0.71) and arousal (M = 4.2 ± SD = 1.18).

In Table 3, we only reported significant correlations between the three affective dimensions of arousal, dominance, and valence and the neuropsychological data. In particular, a positive correlation was found between valence and executive functions (TMT—part B: r = 0.350, *p* < 0.05; TMT B-A: r = 0.364, *p* < 0.05). A positive correlation was found between arousal and both apathy (AES: r = 0.350, *p* < 0.05) and anxiety (HARS: r = 0.401, *p* < 0.05). A negative correlation was found between dominance and all mood scales (AES: r = − 0.411, *p* < 0.05; BDI: r = − 0.420, *p* < 0.05; and HARS: r = − 0.523, *p* < 0.01).

## Discussion

This is the first study to examine the emotional impact of the pandemic, about 20 months after the outbreak, on healthy older people using a unique interdisciplinary approach. The latter combines neuropsychological data and recordings of emotional dysregulation using fEMG and SAM during the presentation of pandemic-related images. Although it is important to emphasize that correlation does not imply causation, our study can help to explain how emotional stimuli, i.e. images from the pandemic context, trigger fEMG responses measured by mean amplitude, and how these are related to multidimensional neuropsychological data.

Our results showed that increased corrugator activity was associated with decreased performance on cognitive tests assessing global cognition, long-term memory, visual processing, and executive functions. In addition, a positive correlation was found between increased corrugator activity and longer walking time on a linear path, i.e., slower gait speed. On the other hand, we found a positive correlation between increased zygomatic activity and mood changes, i.e. higher levels of apathy, depression, and anxiety, while no significant correlations were found between fEMG-1 and mood scales.

In terms of neuropsychological data and SAM, we found an association between a) poorer executive performance (i.e. longer times on TMT – part B and B-A) and more positive valence ratings; b) higher scores for apathy (AES) and anxiety (HARS) and higher levels of arousal; c) higher scores for apathy, depression (BDI) and anxiety and lower control over emotion regulation (dominance).

The results obtained are consistent with a strategy of re-evaluating emotional stimuli in response to the challenges of the pandemic, but no study had been conducted during such a stressful event. Interestingly, COVID-19-related stimuli triggered the activation of bottom-up affectivity strategies associated with higher mood levels and interacted with top-down factors that played an important role in the dysregulation of cognitive control, namely reappraisal. A recent meta-analysis of the literature revealed that fEMG activity of the corrugator generally corresponds with changes in valence and significantly decreases during reappraisal of negative emotions^36^. In agreement with these results, we found that higher positive valence of COVID-19 images was associated with poorer performance, i.e., longer TMT response time, and that an increase in fEMG-1 activity was associated with inadequate reappraisal. This could be due to a slowdown in cognitive control abilities associated with executive tasks such as the TMT. Indeed, the TMT is useful to assess selective attention, response inhibition, and monitoring processes that impact emotion regulation in terms of reappraisal. Interestingly, the correlation between increased corrugator activity and slowed gait speed reinforces and complements the previously observed association with poorer TMT executive performance in terms of information processing speed and cognitive flexibility in cognitively preserved older adults^37^.

The results of our study show an unexpected and counterintuitive positive correlation between higher zygomatic activity and higher levels of apathy, depression, and anxiety. Indeed, this type of contraction pattern is usually associated with facial expressions of positive emotions, such as smiling^16^. However, other evidence suggests that the role of this muscle activity in the expression of emotion is less stereotyped and that the modulators of its use are not yet fully understood. For example, fEMG activation of the zygomaticus during neutral and negative emotions is ambiguous, as the position of the zygomaticus major is close to several muscles such as the zygomaticus minor, masseter, and buccinators^6^, which are associated with negative emotions (e.g., frustration and skepticism)^38^. In addition, fear and anger triggered greater zygomaticus muscle activity, reflecting a so-called “miserable smile”^39^.

Considering that mood changes are controlled by bottom-up processes, anxiety increases attention to threatening stimuli and impairs the attentional control system^40^. Indeed, our results seem to suggest that the effect of higher mood levels influences cognitive abilities such as attention and memory, in terms of higher demands on cognitive emotion regulation during reappraisal. Although no associations were observed between zygomatic activity and executive functions, we found that higher levels of apathy, depression and, more significantly, anxiety were associated with lower dominance, while higher levels of apathy and anxiety were associated with higher arousal. These associations contribute to the previous observation that executive functions support emotion regulation^41^ during reappraisal by promoting the processing of COVID-related stimuli in novel ways.

In addition, the observed positively oriented pattern of facial muscle contraction in response to pandemic-related stimuli could be another aspect that should be discussed in the context of the well-known “positivity effect”^42^ in healthy aging. It is hypothesized that this effect enables the maintenance of emotional functions despite cognitive and physical decline^43–46^. If mimic reactivity is a medium of emotional expression, changes in its expression with age could be a means of maintaining emotional self-regulation in healthy older people^47^. Therefore, the above-mentioned reactivity may have contributed to shaping facial expressions as a means of emotional dysregulation through a defensive strategy of reappraisal during the pandemic period.

The study provides a new and comprehensive perspective on emotional dysregulation through a unique approach that combines neuropsychological assessments with facial muscle reactivity data that allow a better understanding of the emotional impact of the pandemic. We used visual stimuli that are timely and relevant given the ongoing impact of the pandemic and the fact that subjects were experiencing a critical health crisis. Our results show how the pandemic impacted healthy older adults approximately 20 months after the outbreak, in terms of a possible neuro-psycho-physiological signature of emotional dysregulation. Future studies could extend these data and contribute to the generalizability of the obtained results by investigating other measures of emotional (dys)regulation and the factors influencing facial reactivity, thus helping to develop new interventions for the emotional well-being of older people during stressful events. Further research would be needed to confirm and extend our findings, also considering other important aspects. For example, a cross-sectional approach could allow the identification of differences between younger and older people to (1) differentiate baseline values, (2) compare the effects of emotion regulation, and (3) clarify the conditions under which older people’s facial expressions are reduced or enhanced when processing visual pandemic stimuli. Finally, the use of dynamic stimuli, such as movies, would likely have favored stronger emotional activation, as found in some psychophysiological studies (e.g.,^48,49^).

## Methods

### Participants

Older adults attending courses on the neuropsychology of healthy and active aging at the University of the Third Age in Turin (N = 200) were proposed for participation in the study.

The minimum sample size, approximately 20% of the total sample, i.e., 35 socially active subjects who did not complain of SCD, were recruited voluntarily, according to the inclusion criteria and the results of an a priori power analysis performed with G*Power^50^, based on the effect size of a previous study^47^. Specifically, Pedder et al. analyzed facial reactivity in 35 older adults and reported significant zygomaticus (*p* < 0.001, Cohen’s d = 1.26) and corrugator (*p* < 0.001, Cohen’s d = 1.33) activity during passive viewing of images with positive or negative valence^47^. The power analysis yielded a minimum sample size of 26 and 24 participants to detect significant zygomaticus and corrugator reactivity, respectively, with α = 0.001 and power (1−β) = 0.99.

From June to December 2021 (i.e., 15–21 months after the outbreak of the pandemic), these subjects (27 women; 62–82 years) agreed to undergo an in-depth neuropsychological examination to assess their cognitive functioning, mood, and physical health.

Participants also took part in a psychophysiological examination to assess emotional reactivity to images related to the COVID-19 pandemic.

Subjects were excluded if they: were < 60 years old and thus could not be categorized as “older adults”^51^; complained of SCD^25^; had neurological or psychiatric conditions (CIRS^35^). Informed consent was obtained for all subjects to participate in the study. The experiment was designed and conducted in accordance with the ethical guidelines of the University of Turin and was approved by the Ethics Committee (Prot. No. 151786).

### Multidimensional neuropsychological battery

The multidimensional neuropsychological battery assessed cognitive performance, mood, and physical and health status. To avoid fatigue, the neuropsychological test was divided into two sections, each lasting around 45 min and conducted on the same day.

#### Socio-demographic characteristics and cognitive domain

We assessed participants’ SES using the four-factor index of social status^20,21^, i.e., HI, which combines educational level, gender, occupation, and marital status to determine the social status of individuals or families.

The possible presence of SCD was assessed using the Jessen criteria^25^ and the CFI^26^, which includes both self and partner reports to provide a more accurate measure. Global cognition was assessed using the ACE-R^22^, which also includes the MMSE score^23^. Executive function was assessed using the MoCA^27^ and the TMT—parts A and B^29^. The TMT—part A was used to assess information processing speed (i.e., psychomotor speed). Speech comprehension was assessed with the TT^30^, while immediate and delayed recall were analyzed with the RMT^28^.

#### Mood domain

The mood domain was assessed using the following scales: AES^31^, BDI^32^, and HARS^33^, to objectify apathy, depression, and anxiety, respectively.

#### Physical and health status domain

We used the phenotypic frailty model^34^ to assess physical and health status. Five criteria were considered: a) weight loss, b) grip strength, c) self-reported fatigue, d) decreased walking speed, and e) decreased physical activity. Based on the presence of the five criteria, participants could be categorized as robust (none), pre-frail (1 or 2), or frail (3 or more).

Information on clinical history, symptoms, current illnesses, and pharmacotherapy was collected using CIRS^35^. Specifically, we reported the mean and standard deviation of the CIRS—severity and comorbidity indices.

Participants were asked about their health status, in particular (a) whether they had been vaccinated against SARS-CoV-2 (and how many doses had been administered) and (b) whether they had previously reported an infection with SARS-CoV-2.

### Psychophysiological evaluation

The participants were welcomed and introduced to the experiment. They were then asked to sit in a comfortable chair in the observation laboratory of the Department of Psychology. During the experiment, they were instructed to focus on images randomly presented on a 17.3-inch monitor that reminded them of the most critical phase of the pandemic. They were also instructed to rate their emotional state using the SAM^17^, as recommended by Lang et al.^52^, after each image was shown. The psychophysiological assessment was conducted a few days after the neuropsychological assessment and lasted between 45 and 60 min.

The images of the COVID-19 pandemic, ranging from daily life to hospital contexts, were previously selected by our group and recently used by to assess HRV as an indicator of emotional (dys)regulation in terms of dynamic changes in the autonomic nervous system^3^. Specifically, 124 images related to the COVID-19 pandemic were selected via the website “Google images” (https://images.google.com/ (accessed July 1, 2022) using the keyword “COVID”.

To select the images that met certain inclusion criteria, they were evaluated individually by all authors. The images should (i) show people of different ages, genders, and socio-demographic backgrounds; (ii) have elements relevant to the pandemic context such as masks, gloves, and medical staff; (iii) depict typical COVID-19 everyday situations and hospital contexts; (iv) show at least two people interacting (“social” images); and (v) show people with exclusively Caucasian facial features (“ethnicity”) to increase the familiarity of the participants. To avoid overlap, only unique images with different content, formats, and resolutions were considered. Disagreements that arose at this stage were resolved through discussion between all authors. Cohen’s K was used to assess the level of agreement according to the predefined eligibility criteria: % agreement 100; Cohen’s K: 1.

At the end of the assessment phase, 75 images met the inclusion criteria. As the study was conducted between the first and second Italian lockdown (late 2020 to early 2021), images related to COVID-19 were selected to increase the perceived personal and emotional relevance and thus potentially elicit a wider range of emotional responses.

Each image was rated by the participants according to affective dimensions using SAM, an instrument commonly used in psychological research to measure emotional reactions and subjective feelings^17^. SAM consists of a series of three drawings of human figures, i.e., manikins, each representing a specific emotional state (dimensions): Arousal (calm to excited), Dominance as the degree of control that a stimulus triggers (controlled to controlling), and Valence (negative to positive). For each dimension, the subjects had to rate the intensity that best corresponded to their emotional state on a 9-point Likert scale (1 = lowest intensity; 9 = highest intensity).

For all participants, we considered the mean ± standard deviation of all presented images in terms of arousal, dominance, and valence.

### EMG recording and signal processing

Facial EMG activities were recorded using 4 mm diameter bipolar silver/silver chloride (Ag/AgCl) skin surface electrodes and a gel-filled recording chamber. The bipolar electrodes were placed on the corrugator supercilii (fEMG-1) and zygomaticus major muscle (fEMG-2) areas on the left side of the participants’ face according to the EMG placement guidelines recommended by Fridlund and colleagues^53^.

A 2-min baseline session was then conducted with all participants to establish a stable reference value. Psychophysiological assessment began after a specific trigger synchronized with the experimental stimuli. Finally, after a debriefing session, the experimenter helped the participants to remove all electrodes and patches.

The EMG signals were recorded at a sampling rate of 1024 Hz (1024 per second) to ensure sufficient variability for signal processing of this complex muscle activity.

The raw EMG signals were filtered with a 50 Hz notch filter to reduce power noise and with a 10 Hz high-pass filter to exclude noise that could be caused by the participants’ movements. The EMG signals were then displayed on a high-resolution computer monitor to manually exclude stimulus-independent artifacts (e.g., due to coughing or voluntary pouting) by visual inspection.

The main measure used in the study was the amplitude of the EMG signal, which reflects the level of muscle activity, while participants viewed images with high ecological validity as pandemic scenarios that were familiar to them and that they had just experienced. Higher amplitude values indicate greater muscle activation, suggesting increased emotional responsiveness to stimuli.

By examining the amplitude of EMG signals in response to emotional stimuli and recording facial expressions, we aimed to objectify the relationship between positive or negative emotion regulation and the variables assessed with the multidimensional neuropsychological battery.

Signals were recorded using Nexus-4 and then processed using custom software in MATLAB 7.10.0 (R2010a) (The Mathworks, Inc; Natick, MA, USA). The responses were then processed using specialized software developed with MATLAB 9.13.0 (R2022b) (The Mathworks, Inc., Natick, MA, USA).

### Statistical analysis

The Jamovi Statistics software (version 2.2.5.0) and RStudio (version 2023.06.0 + 421) were used to perform the analyses. Kolmogorov–Smirnov and Shapiro–Wilk normality tests were performed to check whether all variables were normally distributed.

To examine the relationship between emotional (dys)regulation, as indexed by the mean amplitude of corrugator activity (fEMG-1) or zygomatic activity (fEMG-2), and neuropsychological data, we calculated a correlation matrix using Pearson’s “r” correlation coefficient.

In addition, we also examined the relationship between the affective dimensions using SAM and the neuropsychological data in the correlation matrix.

Confidence intervals were included in the correlation matrices to ensure that they did not contain zero and to provide an additional indication of the significance of the results.

## Data Availability

The datasets generated and/or analyzed in the current study are available upon reasonable request from the corresponding author.
